# The Impact of Environmental Mn Exposure on Insect Biology

**DOI:** 10.3389/fgene.2018.00070

**Published:** 2018-03-01

**Authors:** Yehuda Ben-Shahar

**Affiliations:** Department of Biology, Washington University in St. Louis, St. Louis, MO, United States

**Keywords:** manganese, manganism, dopamine, *Drosophila melanogaster*, *Apis mellifera*, insects, arthropoda

## Abstract

Manganese (Mn) is an essential trace element that acts as a metal co-factor in diverse biochemical and cellular functions. However, chronic environmental exposure to high levels of Mn is a well-established risk factor for the etiology of severe, atypical parkinsonian syndrome (manganism) via its accumulation in the basal ganglia, pallidum, and striatum brain regions, which is often associated with abnormal dopamine, GABA, and glutamate neural signaling. Recent studies have indicated that chronic Mn exposure at levels that are below the risk for manganism can still cause behavioral, cognitive, and motor dysfunctions via poorly understood mechanisms at the molecular and cellular levels. Furthermore, in spite of significant advances in understanding Mn-induced behavioral and neuronal pathologies, available data are primarily for human and rodents. In contrast, the possible impact of environmental Mn exposure on brain functions and behavior of other animal species, especially insects and other invertebrates, remains mostly unknown both in the laboratory and natural habitats. Yet, the effects of environmental exposure to metals such as Mn on insect development, physiology, and behavior could also have major indirect impacts on human health via the long-term disruptions of food webs, as well as direct impact on the economy because of the important role insects play in crop pollination. Indeed, laboratory and field studies indicate that chronic exposures to metals such as Mn, even at levels that are below what is currently considered toxic, affect the dopaminergic signaling pathway in the insect brain, and have a major impact on the behavior of insects, including foraging activity of important pollinators such as the honey bee. Together, these studies highlight the need for a better understanding of the neuronal, molecular, and genetic processes that underlie the toxicity of Mn and other metal pollutants in diverse animal species, including insects.

## Introduction

Manganese (Mn) is an essential trace element that acts as a metal co-factor in diverse biochemical and cellular functions (Mertz, 1981; Santamaria and Sulsky, 2010). However, chronic environmental or occupational exposures to high levels of Mn are often neurotoxic, and a well-established risk factor for severe, atypical parkinsonian syndrome (manganism) in humans (Lucchini et al., 2009; Racette, 2014; Dorman, 2017). The exact cellular and molecular mechanisms that mediate the specific neurotoxic effects of Mn exposure to neuronal pathways associated with motor and cognitive functions are still not well understood. However, studies in humans, and in primate and rodent animal models suggest that chronic Mn exposure leads to its accumulation in the basal ganglia, pallidum, and striatum regions of the mammalian brain, with subsequent neurotoxic effects on the dopaminergic, GABAergic, and glutamatergic signaling pathways (Olanow, 2004; Fordahl et al., 2010; Peneder et al., 2011; Karki et al., 2013; Sidoryk-Wegrzynowicz and Aschner, 2013). Surprisingly, more recent studies have indicated that chronic exposures to Mn at levels that are below the known risk threshold for manganism, could still cause behavioral, cognitive, and motor dysfunctions in humans, non-human primates, and rodents (Schneider et al., 2006; Claus Henn et al., 2010; Al-Lozi et al., 2017). Yet, whether these effects are mediated by the same neuroanatomical regions and neural signaling pathways that induce manganism remains somewhat unresolved (Gwiazda et al., 2002; Khalid et al., 2011; Li et al., 2017).

Although much of the research focus on metal biology in general, and Mn neurotoxicity in particular, has been in mammalian models, emerging data indicate that invertebrate species such as the worm *C. elegans* and insects such as the fruit fly *Drosophila melanogaster*, the honey bee *Apis mellifera*, and others are also highly sensitive to Mn toxicity, with negative impacts on behavior and higher brain functions (Orgad et al., 1998; Ben-Shahar et al., 2004; Mogren and Trumble, 2010; Chen et al., 2015a,b; Horning et al., 2015; Søvik et al., 2015, 2017). Nonetheless, in spite of significant advances in understanding the direct impact of environmental Mn exposure on human health, its environmental impact on neural functions and behavior of other animals, especially invertebrates, remains mostly unexplored. Consequently, the long-term effects of environmental Mn on food webs via its impact on the physiology of plants and pollinators are likely to play an important role in the health of ecosystems (Gall et al., 2015).

## Manganese and Insect Biology

Like vertebrates, all insects require Mn as a metal co-factor for the catalytic actions of diverse enzymes (Burnell, 1988; Schramm, 2012), including the universal mitochondrial Mn-superoxide dismutase (Holley et al., 2012). In addition to its more general role as an enzymatic metal co-factor, Mn also plays a direct role in various molecular and physiological processes specifically associated with insect development and behavior. One of the first studies to describe the effect of Mn on insect physiology was the description of increased melanism in moth larvae exposed to food laced with Mn (Harrison, 1928). Later studies of the chemical composition of the insect cuticle revealed that in some species, Mn and zinc (Zn) constitute up to 10% of the total dry weight of the cuticle (Eric Hillerton et al., 1984; Quicke et al., 1998; Morgan et al., 2003; Broomell et al., 2008). Studies of the process of cuticle sclerotization in these species revealed that Mn and Zn are required for the formation of mechanically hard cuticular regions in the ovipositor of females, the female organ used for egg laying, which helps them penetrate hard plant materials such as fruit skins, as well as for development of the abrasion-resistant cutting edge of chewing mandibles in insects that feed on hard-to-chew foods such as dry seeds (Eric Hillerton et al., 1984; Cribb et al., 2008; Andersen, 2010).

Although essential, chronic dietary exposure to Mn is often toxic and detrimental to the fitness of most arthropod species, including insects, via effects on embryonic development, feeding behaviors, reproduction, immunity, and general survivability (Olsén, 2011; Kula et al., 2014; Ternes et al., 2014; de Barros et al., 2017; Martinek et al., 2018). For example, the collembolan *Folsomia candida* is highly sensitive to Mn in its diet (Kuperman et al., 2004). However, other arthropods, such as the fly *Megaselia scalaris*, show few adverse effects of Mn exposure, even at levels as high as 2,600 mg Mn/kg (Sorensen et al., 2009). While the exact mechanisms that affect the sensitivity threshold of insects to Mn exposure are mostly unknown, studies suggest that some species can actively avoid the consumption of Mn-contaminated foods (Rokytova et al., 2004), while others evolved mechanisms for efficient excretion of dietary Mn and/or its sequestration in specific body parts (Kula et al., 2014; Martinek et al., 2017, 2018).

To date, the majority of formal environmental risk assessments of toxic exposures to metals such as Mn have been characterized in the contexts of inhaled particles under specific human occupational conditions and practices, or controlled laboratory inhalation exposure studies by using mammalian animal models (Tjalve and Henriksson, 1999; Dorman et al., 2002; Antonini et al., 2006; Elder et al., 2006; Erikson et al., 2007; Bailey et al., 2017; Bevan et al., 2017). However, emerging data indicate that metal exposure via drinking water and its accumulation in aquatic environments represents a considerable risk as well (Kavcar et al., 2009; Bouchard et al., 2011). Yet, the possible broader ecological neurotoxic impact of Mn exposure either via inhaled or oral pathways on animal physiology, behavior, and overall fitness, remains mostly unknown.

Evaluating true environmental risks for insects is further complicated by of the microscale features of their ecological niches, and their diverse feeding ecologies and complex life cycles. Nevertheless, although the direct sources of Mn accumulation in aquatic and terrestrial environments are often unknown, geographical proximity to anthropogenic activities associated with metal mining and processing, and the commercial use of Mn-containing fertilizers, is a well-established risk factor. Subsequently, because the salt forms of Mn, and similarly toxic metals, are often water soluble, they readily enter food chains via their accumulation in both marine and fresh water environments. Not surprisingly, several studies have found that aquatic insects exhibit rapid uptake and tissue accumulation of Mn and other divalent metal ions present in their environment (Poteat et al., 2012), which often have a direct negative effect on their fitness (Hernroth et al., 2004; Krång and Rosenqvist, 2006; Oweson et al., 2008). Although it is reasonable to assume that, due to its solubility, the toxic impact of Mn on insects and other invertebrates is primarily restricted to aquatic environments; data suggest that Mn exposure of insects with an aquatic larval stage could still carry fitness costs in the terrestrial adult phase. Furthermore, insects with a complex life cycle could bridge the negative impacts of metal exposure across the aquatic and terrestrial ecosystems and their associated food webs (Custer et al., 2008; Dittman and Buchwalter, 2010; Kraus et al., 2014).

Natural and anthropogenic sources of environmental Mn could also have direct negative impacts on terrestrial insects. Because many insects seem to have no aversive behavioral response to the presence of Mn and other metals in their food (Mogren and Trumble, 2010), one possible oral path to exposure is presented by the accumulation of specific metals in plants and their subsequent consumption by phytophagous insects (Devkota and Schmidt, 2000; Rodrigues et al., 2008). These exposure risks are further amplified in insect pollinators, which seem to be highly sensitive to metals (Ben-Shahar et al., 2004; Moroń et al., 2012; Vanbergen and Initiative, 2013; Søvik et al., 2015), most likely via the consumption of nectar and pollen by adult insects (Behmer et al., 2005), as well as throughout development in bee species that provision their larvae with pollen and nectar (Somerville and Nicol, 2002; Moroń et al., 2014). Because metals can accumulate in the nectar of flowering plants, insect pollinators seem to be especially sensitive to environmental metals, including Mn (Haarmann, 1998; Meindl and Ashman, 2013; Søvik et al., 2015). This particular concern is alarming because of the increase in global pollinator duress due to the negative pressure of various pathogens (Cox-Foster et al., 2007; Naug, 2014), parasites (Martin et al., 2012), and possibly insecticides (Woodcock et al., 2016; McArt et al., 2017), which together lead to major costs in pollinator fitness, which could carry major economic and ecological consequences (Khoury et al., 2011). Metal toxicity is further amplified in social pollinators, such as the honey bee, which consume nectar in the form of concentrated honey, which leads to a significant accumulation of contaminants such as heavy metals in both honey and bee tissues via prolonged exposure throughout development (Leita et al., 1996; Hladun et al., 2016; Herrero-Latorre et al., 2017; Klein et al., 2017).

## Manganese and Insect Behavior

The recognition that excessive exposure to metals such as Mn could also have an impact on insect neurophysiology and behavior is not new. For example, some of the early laboratory studies of insect muscle physiology revealed that the membrane of these cells is highly permeable to Mn^2+^ and other divalent metal cations, possibly via the action of Ca^2+^ channels (Fukuda and Kawa, 1977). Specifically, several studies demonstrated that increased levels of Mn^2+^ in the extracellular bath significantly reduced the excitability and contractility of visceral muscles in diverse insect species (Deitmer, 1977; Cook and Mark Holman, 1979). However, whether under natural conditions, environmental exposure to Mn negatively affects the fitness of individual insects via its impact on muscle physiology and associated flight-related behaviors remains mostly unexplored.

Exposure to Mn can also have direct effects on the behavior of insects. Although some insect species seem to be able to detect toxic levels of Mn, and therefore behaviorally avoid the consumption of tainted foods, most insects seem to be unable to sense the presence of metals, and some increase the consumption of foods and water that contain harmful levels of some metals (Ben-Shahar et al., 2004; Mogren and Trumble, 2010; Søvik et al., 2015, 2017). Although the direct impact of Mn on insect behavior has been studied in just a few species, data suggest that Mn exposure affects general locomotion, as well as innate behaviors associated with feeding drive and food choices. One of the first clues that Mn might be involved in innate food choices in insects came from forward genetic screens for food choice behaviors in the fruit fly *Drosophila melanogaster*. One of the genes identified is the solute carrier *Malvolio* (*Mvl*), which contributes to the decision of flies to consume high sugar foods (Rodrigues et al., 1995). Subsequently, it was shown that *Mvl* is a divalent metal transporter homologous to the mammalian NRAMP transporters, and that supplementing standard fly food with Mn is sufficient to rescue abnormal food choices in adult flies (Orgad et al., 1998; D’Souza et al., 1999; Southon et al., 2008). Similarly, studies in the honey bee revealed that the brain expression of *Mvl* increases with the age-dependent division of labor exhibited by workers in honey bee colonies, and is associated with age-dependent decrease in the appetitive response threshold to sugar. Furthermore, feeding young bees with Mn resulted in a dose-dependent lowering of their response threshold to sugar, and a precocious transition from in-hive behaviors to foraging (Ben-Shahar et al., 2004). A follow up study revealed that Mn-treated bees were also poor foragers with shorter foraging career than untreated controls, further indicating that Mn exposure could lead to neurodevelopmental and cognitive deficits in pollinators (Søvik et al., 2015). Consequently, studies by us and others have shown that exposure of honey bees and other pollinators to Mn and other toxic metals could affect their behavioral responsiveness to sucrose, foraging activity, and possibly increase their foraging on metal-contaminated nectars due to abnormally low appetitive response thresholds (Ben-Shahar et al., 2004; Hladun et al., 2012, 2013, 2016; Meindl and Ashman, 2013; Søvik et al., 2015). Although the specific molecular and cellular mechanisms that mediate the effects of environmental exposure to Mn on the behavior of insect pollinators remain mostly understudied, we describe some recent insights into the cellular and molecular bases for its effects on the nervous systems of insects.

## Cellular and Molecular Targets of Manganese in the Insect Nervous System

Although the specific molecular and cellular mechanisms by which Mn exposure leads to abnormal behaviors are not completely understood (Racette et al., 2012; Andruska and Racette, 2015), human pathology and laboratory studies in rodent models indicate that environmental or occupational exposure to high levels of Mn are often associated with the symptoms of an atypical parkinsonian syndrome (Chen et al., 2014; Andruska and Racette, 2015). As in the classic Parkinson’s Disease (PD), these studies clearly demonstrate that exposure to high levels of Mn leads to the specific loss of dopaminergic neurons and associated signaling pathways in the mammalian striatum (Chen et al., 2006, 2014, 2015a; Zhao et al., 2009; Andruska and Racette, 2015). However, why Mn is specifically neurotoxic to these neuronal populations is not yet understood. Previous studies indicated that Mn directly interacts with the neurotransmitter dopamine (Parenti et al., 1988; Prabhakaran et al., 2008), which may explain, at least in part, the specificity of Mn neurotoxicity. At the subcellular level, several studies have suggested that Mn could affect dopaminergic neurons by directly interacting with PD-related proteins such as *α-Synuclein* and PARK9 (Gitler et al., 2009). Other studies in mammalian models suggested that Mn exposure leads to dopaminergic cell death via the upregulation of mitochondrial-derived oxidative stress (Kitazawa et al., 2002; Erikson et al., 2004; Ávila et al., 2014). Recently, increased oxidative stress in response to Mn exposure has also been identified in the insect *Drosophila melanogaster* (Mohandas et al., 2017). However, why this mechanism would specifically affect dopaminergic neurons is not clear. Mn is also likely to affect the dopaminergic system indirectly via its impact on the production of reactive oxygen species (ROS) in non-neuronal microglia (Zhang et al., 2009). Other indirect mechanisms include the inhibition of glutamate uptake by astrocytes, which subsequently could lead to dopaminergic excitotoxicity (Karki et al., 2014; Streifel et al., 2014; Sidoryk-Wegrzynowicz and Aschner, 2015), or the possibility of Mn-dependent epigenetic modifications (Tarale et al., 2017). Overall, these findings suggest that chronic exposure to high levels of Mn disrupts dopaminergic signaling, possibly by modulating the homeostatic relationship between excitatory and inhibitory neurotransmission pathways (Sidoryk-Wegrzynowicz and Aschner, 2013).

Similarly to humans and rodents, studies in insect models such as the fruit fly and the honey bee have indicated that chronic exposure to Mn is toxic to flies in a dose-dependent manner (Mohandas et al., 2017). Furthermore, these studies demonstrated that Mn accumulates in brain tissues even at sub-lethal exposure levels (Ben-Shahar et al., 2004; Søvik et al., 2015, 2017). However, in contrast to the effects of exposure to high levels of Mn, chronic exposure to low levels are associated with a surprising increase in brain levels of dopamine in flies and bees, as well as a transcriptional upregulation of the rate-limiting enzymes in the biosynthesis pathways of dopamine (Søvik et al., 2015). A broader analysis of the brain neurogenomic response to chronic Mn exposure, at levels that are sufficient to induce precocious foraging in honey bees, revealed a unique transcriptional response that was different from that induced by other known pharmacological inducers of foraging behavior such as cGMP (Whitfield et al., 2006).

Furthermore, *in vivo* knockdown of the Mn^2+^transporter *Malvolio* specifically in dopaminergic neurons leads to dramatic changes in the neural architecture of the dopaminergic circuit, which can be rescued by feeding flies Mn^2+^ (Søvik et al., 2017). Because the effects of Mn on dopaminergic signaling seem to be conserved across insects and mammals, future genetic studies in *Drosophila* and other insect models will likely reveal important mechanistic insights into the impact of Mn exposure on neurobiological functions across animals, including humans. Together, the findings that Mn-induced changes in insect brain gene expression patterns represent a unique transcriptional network indicate that the effects of Mn on neuronal and behavioral phenotypes is not due to general neurotoxicity (Sinha et al., 2006).

## Conclusion

Emerging data indicate that although high and low levels of chronic Mn exposure can lead to negative neurological and behavioral outcomes associated with dopaminergic functions, the cellular and molecular mechanisms that mediate their respective phenotypic outcomes are very different. Nonetheless, whether environmental exposure to Mn represents a broad and acute environmental risk for insects and related ecological networks remains mostly anecdotal. Yet, it is very unlikely that insects in affected habitats would not be negatively impacted by the presence of metals. Therefore, further studies of the impact of metals such as Mn on the behavior, physiology, and neural functions of insects would serve several important functions. First, although the specific molecular and cellular processes that are affected by the disruption of Mn homeostasis in the insect brain remain poorly understood, the apparent specific effects of both Mn depletion and saturation on dopaminergic signaling and its associated behavioral phenotypes suggest that this specific, highly conserved neuromodulatory network is key for understanding the role Mn is playing in regulating brain functions and behavior in health and disease in insects, with direct mechanistic implications for other animals, including humans. Therefore, insect models should be used to improve our general mechanistic understanding of the impact of Mn exposure on neural functions at the cellular and molecular levels. Second, understanding better the effects of Mn on insect behavior and physiology could be used for effective biomonitoring of Mn in affected habitats. Finally, studies of the biological impact of Mn exposure on insect behavior and physiology will help us understand better the broader ecological and environmental costs associated with anthropogenic environmental accumulation of metals, and their indirect impact on human society and health by negatively affecting insect pollinators and other important nodes of key food webs.

## Author Contributions

The author confirms being the sole contributor of this work and approved it for publication.

## Conflict of Interest Statement

The author declares that the research was conducted in the absence of any commercial or financial relationships that could be construed as a potential conflict of interest.
